# What proportion of patients with psychosis is willing to take part in research? A mental health electronic case register analysis

**DOI:** 10.1136/bmjopen-2016-013113

**Published:** 2017-03-09

**Authors:** Rashmi Patel, Sherifat Oduola, Felicity Callard, Til Wykes, Matthew Broadbent, Robert Stewart, Thomas K J Craig, Philip McGuire

**Affiliations:** 1 Department of Psychosis Studies, King's College London, Institute of Psychiatry, Psychology & Neuroscience, London, UK; 2 King's College London, Health Service and Population Research, Institute of Psychiatry, Psychology & Neuroscience, London, UK; 3 Department of Geography and Centre for Medical Humanities, Durham University, Durham, UK; 4 Department of Psychology, King's College London, Institute of Psychiatry, Psychology & Neuroscience, London, UK; 5 South London and Maudsley NHS Foundation Trust, Biomedical Research Centre Nucleus, Mapother House, London, UK; 6 Department of Psychological Medicine, King's College London, Institute of Psychiatry, Psychology & Neuroscience, London, UK

**Keywords:** CRIS, C4C, consent, bipolar disorder, clinical research

## Abstract

**Objective:**

The proportion of people with mental health disorders who participate in clinical research studies is much smaller than for those with physical health disorders. It is sometimes assumed that this reflects an unwillingness to volunteer for mental health research studies. We examined this issue in a large sample of patients with psychosis.

**Design:**

Cross-sectional study.

**Setting:**

Anonymised electronic mental health record data from the South London and Maudsley NHS Foundation Trust (SLaM).

**Participants:**

5787 adults diagnosed with a psychotic disorder.

**Exposure:**

Whether approached prior to 1 September 2014 for consent to be approached about research participation.

**Main outcome measures:**

Number of days spent in a psychiatric hospital, whether admitted to hospital compulsorily, and total score on the Health of the Nation Outcome Scale (HoNOS) between 1 September 2014 and 28 February 2015 with patient factors (age, gender, ethnicity, marital status and diagnosis) and treating clinical service as covariates.

**Results:**

1187 patients (20.5% of the total sample) had been approached about research participation. Of those who were approached, 773 (65.1%) agreed to be contacted in future by researchers. Patients who had been approached had 2.3 fewer inpatient days (95% CI −4.4 to −0.3, p=0.03), were less likely to have had a compulsory admission (OR 0.65, 95% CI 0.50 to 0.84, p=0.001) and had a better HoNOS score (β coefficient −0.9, 95% CI −1.5 to −0.4, p=0.001) than those who had not. Among patients who were approached, there was no significant difference in clinical outcomes between those agreed to research contact and those who did not.

**Conclusions:**

About two-thirds of patients with psychotic disorders were willing to be contacted about participation in research. The patients who were approached had better clinical outcomes than those who were not, suggesting that clinicians were more likely to approach patients who were less unwell.

**Strengths and limitations of this study**

- Our study investigates a large sample of patients with psychotic disorders to determine the proportion who consented to be contacted to participate in clinical research. We found that most patients were willing to be contacted but that clinicians tended to approach patients who were less unwell.
- As this was an observational study, it was not possible to determine the explanation for observed variation in rates of consent which are likely to depend on a combination of patient and healthcare service-related factors.
- Our study may have been affected by selection bias as clinicians may have approached patients who were perceived to be more likely to provide consent to contact for research participation.

## Introduction

Patient recruitment is often cited as a challenge in conducting research in mental health, particularly in people with psychotic disorders.^1–3^ The National Institute for Health Research (NIHR) performance report for 2013/2014 showed that only five mental healthcare providers were among the top 100 performing healthcare providers recruiting to clinical research.^4^ One potential contributing factor is the marked discrepancy in research funding for mental and physical healthcare. In the UK, only £9.75 of mental health research funding is invested for each affected individual, compared with £1571 for cancer research funding.^5^ Furthermore, in physical healthcare, patients who participate in research, or who are treated in healthcare organisations that take part in clinical research, are likely to have better clinical outcomes.^6^ ^7^ For example, a recent systematic review^7^ found that patients at centres that conducted clinical trials had better outcomes than patients treated at centres not involved in research.

Patient involvement is an integral part of mental health research.^8^ Indeed patient involvement has gained momentum in mental health research over the years as a number of studies have examined the association of user participation and benefit for research. Ennis and Wykes^9^ found that research studies that involved patients in study design and implementation were more likely to meet their recruitment target. Goodwin *et al*^10^ highlighted the importance of patient input in a study developing an electronic patient reporting application. Other studies have considered patient attitude towards engaging in research reporting facilitators and barriers such as altruism, reassurance about confidentiality, access to study centre, ethnic minority status, stigma and distrust in mental healthcare services.^11–13^

Despite these advances, it is unclear whether research participation among people with mental disorders contributes to better clinical outcomes (as seen in physical healthcare) or if staff are more likely to approach patients about research if they have a relatively good prognosis.

Electronic health records can play a vital role in improving access to clinical research opportunities through consent for contact (C4C) programme whereby clinicians may ask their patients whether they might be interested in being contacted about relevant research opportunities and, if so, give permission to be invited to participate in research.^14–16^

The first aim of the present study was to determine the proportion of patients with psychotic disorders who were willing, in principle, to participate in research. The second objective was to assess whether patients who were approached about research were different to those who were not approached, in terms of their demographic and clinical features, and clinical outcomes. We addressed these issues by accessing an electronic database which contained the clinical records from a large sample of patients with a psychotic disorder. We have chosen to focus on patients with a diagnosis of a psychotic disorder because evidence suggests that there are challenges engaging this particular group in research.^17^

## Methods

### Study setting and participants

The study was conducted using clinical data from the pseudonymised electronic health records of patients receiving mental healthcare from the South London and Maudsley NHS Foundation Trust (SLaM). SLaM provides mental healthcare for the residents of Lambeth, Southwark, Croydon and Lewisham in London, with a total population of 1.5 million. Services for patients with psychotic disorders comprise 74 community and inpatient teams.

The present study analysed data from adults (aged over 16 years) diagnosed with a psychotic disorder and receiving care from in SLaM between 1 September 2014 and 28 February 2015. Diagnosis of a psychotic disorder was defined according to the International Classification of Diseases (ICD)-10 classification system and included schizophrenia or related disorders (schizophrenia (F20), delusional disorder (F22), schizophrenia-like disorders (F23, F28, F29)), schizoaffective disorder (F25), mania or bipolar disorder with psychotic symptoms (F30, F31), psychotic depression (F32.3, F33.3), drug-induced psychosis (F1x.5) and any other psychotic disorder not otherwise specified. Using these criteria, data from 5787 people were obtained and included in the present study.

### Source of clinical data

Data were obtained from the SLaM Biomedical Research Centre (BRC) Case Register,^18^ ^19^ a large data set containing pseudonymised clinical data of over 270 000 patients derived from the electronic Patient Journey System (ePJS). ePJS is an electronic health record system which has been used in all SLaM clinical services since April 2006. Healthcare professionals use ePJS to document all clinical information. The clinical information documented includes structured fields (for demographic information) and de-identified unstructured free-text fields from case notes and correspondence. Data for this study were obtained from these sources of clinical data using the Clinical Record Interactive Search tool (CRIS). CRIS is a bespoke database search and assembly tool which has supported a range of studies^20–25^ using clinical data from the SLaM BRC Case Register.

### C4C programme

The SLaM C4C programme was developed to facilitate recruitment of patients into mental health research studies. A full description of programme development, method and implementation are provided in a previous paper.^14^ In summary, the programme relies on the integration of the ePJS system with the SLaM BRC Case Register and CRIS. Taking consent involves healthcare professionals asking patients whether they would be willing to be contacted (at a later date) by a researcher if they were eligible for a research study on the basis of reviewing their anonymised electronic health records. Patients' responses to this question (whether providing or refusing consent) are recorded in ePJS. Researchers who are conducting clinical studies can then use the CRIS tool to search the SLaM BRC Case Register to identify and contact patients who are willing to be approached about participation in a research project.

### Ethical approval and research governance

The SLaM BRC Case Register and CRIS have received ethical approval from the Oxfordshire Research Ethics Committee C (08/H0606/71+5) as a pseudonymised data set for mental health research.^26^ The SLaM C4C model was reviewed and approved by the National Information Governance Board for Health and Social Care (NIGB) Ethics and Confidentiality committee (reference ECC 2-08/2010).^14^ A patient-led oversight committee provides governance for all projects conducted using these data, including those that use the C4C programme. Research studies can only be registered with the C4C programme if they have received approval from a UK Research Ethics Committee. A robust firewall and data security framework governs access to clinical data from the case register and only approved researchers are permitted to access data from the case register.^26^

### Exposure

The exposure was defined as whether or not patients had been approached for consent to participate in research prior to 1 September 2014. This definition was chosen to ensure that the exposure always occurred prior to the measurement of the clinical outcome measures.

### Clinical outcome measures

The primary outcome measure was the number of days spent as an inpatient in a psychiatric hospital in SLaM between 1 September 2014 and 28 February 2015. This represents a robust marker of illness severity from the patients' perspective, as well as its impact on family and carers, and on mental healthcare services. Secondary outcome measures included whether or not the admission had been compulsory, under the UK Mental Health Act,^27^ and the first recorded total score on the Health of the Nation Outcome Scale (HoNOS) recorded between 1 September 2014 and 28 February 2015. HoNOS is a scale of illness severity which is routinely recorded in UK mental healthcare services.^28^ The scale covers 12 domains: overactive, aggressive, disruptive or agitated behaviour; non-accidental self-injury; problem drinking or drug-taking; cognitive problems; physical illness or disability problems; problems associated with hallucinations and delusions; problems with depressed mood; other mental and behavioural problems; problems with relationships; problems with activities of daily living; problems with living conditions; problems with occupation and activities. Each domain is scored on a scale of 0–4 with a maximum possible total score of 48. The total score was chosen as a secondary outcome in order to capture a measure of illness severity that would apply to outpatients as well as inpatients. Complete outcome data were available for the primary outcome measure and compulsory hospital admission. HoNOS score data were available for 3052 (52.7%) of patients included in the study.

### Covariates

The following variables were extracted as categorical covariates for multivariable analyses: age, gender, ethnicity, marital status, diagnosis and the clinical service providing care to the patient. The number of hospital admissions between 1 September 2011 and 31 August 2014 was extracted as a continuous covariate. All categorical covariate data obtained were those recorded closest to 1 September 2014. Ethnicity was recorded according to categories defined by the UK Office for National Statistics.^29^ Marital status was recorded in the following categories: married or cohabiting; divorced or separated; single; unknown. Diagnosis was defined according to the groups described in the *Study setting and participants* section.

### Statistical analysis

Stata (V.12.0) was used to analyse the data (StataCorp. Stata Statistical Software: Release 12. Coll Station, TX: StataCorp LP, 2011). Descriptive statistics for the exposure, outcome and covariate variables were obtained as frequencies and percentages for categorical variables and means and SDs for continuous variables. In order to assess whether there were any patient factors (age; gender; ethnicity; marital status; diagnosis) or mental health service-related factors (clinical service) associated with being approached for (C4C), the associations of patient factors with being approached for consent were tested first individually with univariate binary logistic regression followed by a multivariable binary logistic regression analysis adjusted for patient factors and clinical service. A further analysis on patients who were approached for consent was undertaken to investigate the association between whether or not patients gave consent and patient and mental health service-related factors using the same univariate and multivariable binary logistic regression method.

The associations of being approached for consent with number of inpatient days and with total HoNOS score were tested using multiple linear regression. The association between being approached for consent and compulsory hospital admission was tested using multivariable binary logistic regression. The association of number of hospital admissions between 1 September 2011 and 31 August 2014 with each clinical outcome measure was tested separately in univariate analyses and found to be a significant predictor of all clinical outcomes measures: number of inpatient days β coefficient 6.0 (95% CI 5.4 to 6.6, p<0.001); compulsory hospital admission OR 1.35 (1.28 to 1.42, p<0.001); total HoNOS score β coefficient 0.5 (0.3 to 0.6, p<0.001). For this reason, four regression models were generated for each analysis of association with clinical outcome measures as follows:

- Model 1: unadjusted;
- Model 2: adjusted for number of hospital admissions between 1 September 2011 and 31 August 2014;
- Model 3: adjusted for all factors in model 2 plus age, gender, ethnicity, marital status and diagnosis;
- Model 4: adjusted for all factors in model 3 plus clinical service.

This stepwise approach was chosen in order to first examine the effect of adjusting for number of prior hospital admissions (model 2) before adjusting for patient-related factors (model 3) and then mental health service-related factors (model 4).

A further analysis on patients who were approached for consent was undertaken to investigate the association between whether or not patients gave consent and clinical outcome methods using the same stepwise regression method. Where there was missing data in covariate data (184 patients with no known marital status), the missing data category was included as a predictor variable in regression analyses.

## Results

### C4C and patient and mental healthcare service-related factors

Of the 5787 patients included in the study, 1187 (20.5%) had been approached for C4C to participate in research. Nine hundred and forty-seven patients had been approached in the community and 240 after they had been admitted to hospital. Of those approached, 773 (65.1%) gave consent to be contacted about participation in future research studies.

Table 1 shows the breakdown of who was approached by patient-related factors. Univariate regression analysis showed that patients aged between 46 and 55 years were the most likely to be approached, but that there was no difference between men and women. Black patients were more likely to be approached than white, Asian and other ethnic groups. Patients who were married or cohabiting were less likely to be approached compared with those who were single. Patients with psychotic depression were less likely to be approached compared with patients with schizophrenia or related disorders. Patients with a greater number of admissions between 1 September 2011 and 31 August 2014 were more likely to be approached. However, after adjusting for all factors in a multivariable analysis, there was no longer a significant difference in being approached among patients with demographic factors or between different diagnostic groups.

**Table 1:** Binary logistic regression analysis of patient and service-related factors associated with being approached for C4C, n=5787

Factor	Group	Number in sample	Percentage approached for consent (%)	Unadjusted OR	95% CI, p value	Adjusted OR*	95% CI, p value
Age	Age 16–25 years	726	18.9	Reference		Reference	
	26–35	1134	19.9	1.07	0.84 to 1.36, p=0.57	0.75	0.58 to 0.98, p=0.03
	36–45	1225	19.8	1.06	0.84 to 1.34, p=0.63	0.64	0.48 to 0.85, p=0.002
	46–55	1360	24.2	1.37	1.10 to 1.72, p=0.006	0.81	0.61 to 1.06, p=0.12
	56–65	687	21.5	1.18	0.91 to 1.53, p=0.21	0.77	0.56 to 1.06, p=0.11
	>65	655	16.0	0.82	0.62 to 1.09, p=0.17	0.74	0.53 to 1.03, p=0.07
Gender	Female	2394	20.5	1.00	0.88 to 1.14, p=1.00	1.00	0.87 to 1.16, p=0.97
	Male	3393	20.5	Reference		Reference	
Ethnicity	White	2602	19.9	Reference		Reference	
	Asian	365	16.2	0.78	0.58 to 1.04, p=0.09	0.85	0.62 to 1.17, p=0.33
	Black	2313	23.4	1.23	1.07 to 1.41, p=0.003	0.97	0.83 to 1.13, p=0.69
	Other ethnic group	507	13.8	0.65	0.49 to 0.85, p=0.002	0.69	0.51 to 0.92, p=0.01
Marital status	Married/cohabiting	704	16.5	0.72	0.58 to 0.89, p=0.002	0.75	0.59 to 0.95, p=0.02
	Divorced/separated	518	23.6	1.13	0.91 to 1.40, p=0.28	0.96	0.75 to 1.22, p=0.73
	Single	4381	21.5	Reference		Reference	
	Marital status unknown	184	4.3	0.17	0.08 to 0.34, p<0.001	0.32	0.15 to 0.66, p=0.002
Diagnosis	Schizophrenia and related	3705	20.8	Reference		Reference	
	Schizoaffective	288	24.3	1.22	0.92 to 1.62, p=0.17	1.20	0.88 to 1.64, p=0.25
	Bipolar disorder	366	23.2	1.15	0.89 to 1.48, p=0.29	0.88	0.66 to 1.18, p=0.39
	Psychotic depression	558	15.9	0.72	0.57 to 0.92, p=0.008	0.86	0.66 to 1.12, p=0.26
	Drug-induced psychosis	173	18.5	0.86	0.58 to 1.28, p=0.46	0.95	0.62 to 1.46, p=0.81
	Other psychosis	697	19.9	0.95	0.77 to 1.16, p=0.59	0.93	0.75 to 1.16, p=0.52
Number of admissions between 1 September 2011 and 31 August 2014				1.28	1.22 to 1.34, p<0.001	1.28	1.22 to 1.34, p<0.001

*Adjusted for age, gender, ethnicity, marital status, diagnosis, number of admissions between 1 September 2011 and 31 August 2014, and clinical service.

C4C, consent for contact.

A further analysis of the patients who were approached for consent to examine the association of whether patients gave consent with patient factors (table 2) demonstrated that patients aged between 16 and 25 years were most likely to give consent compared with other age groups. The factor most strongly associated with giving consent was if patients were approached while they were in hospital rather than as outpatients.

**Table 2:** Binary logistic regression analysis of patient and service-related factors associated with whether consent was given, n=1187

Factor	Group	Number in sample	Percentage giving consent (%)	Unadjusted OR	95% CI, p value	Adjusted OR*	95% CI, p value
Age	Age 16–25 years	137	81.0	Reference		Reference	
	26–35	226	73.5	0.65	0.39 to 1.09, p=0.10	0.98	0.54 to 1.77, p=0.94
	36–45	242	65.3	0.44	0.27 to 0.73, p=0.001	0.72	0.40 to 1.32, p=0.29
	46–55	329	60.5	0.36	0.22 to 0.58, p<0.001	0.62	0.34 to 1.11, p=0.11
	56–65	148	63.5	0.41	0.24 to 0.70, p=0.001	0.65	0.34 to 1.26, p=0.20
	>65	105	42.9	0.18	0.10 to 0.31, p<0.001	0.28	0.14 to 0.55, p<0.001
Gender	Female	491	64.4	0.94	0.74 to 1.20, p=0.64	0.86	0.65 to 1.13, p=0.27
	Male	696	65.7	Reference		Reference	
Ethnicity	White	517	66.5	Reference		Reference	
	Asian	59	71.2	1.24	0.69 to 2.25, p=0.47	1.11	0.58 to 2.12, p=0.75
	Black	541	63.0	0.86	0.67 to 1.10, p=0.23	0.73	0.55 to 0.97, p=0.03
	Other ethnic group	70	65.7	0.96	0.57 to 1.63, p=0.89	0.91	0.51 to 1.62, p=0.74
Marital status	Married/cohabiting	116	62.9	0.88	0.59 to 1.32, p=0.54	1.21	0.77 to 1.91, p=0.41
	Divorced/separated	122	60.7	0.80	0.54 to 1.18, p=0.26	1.05	0.67 to 1.65, p=0.83
	Single	941	65.8	Reference		Reference	
	Marital status unknown	8	87.5	3.64	0.45 to 29.7, p=0.23	2.88	0.30 to 27.6, p=0.36
Diagnosis	Schizophrenia and related	772	62.7	Reference		Reference	
	Schizoaffective	70	62.9	1.01	0.61 to 1.67, p=0.98	1.08	0.62 to 1.87, p=0.80
	Bipolar disorder	85	68.2	1.28	0.79 to 2.06, p=0.32	1.03	0.61 to 1.75, p=0.91
	Psychotic depression	89	75.3	1.81	1.10 to 3.00, p=0.02	1.60	0.92 to 2.77, p=0.10
	Drug-induced psychosis	32	84.4	3.21	1.22 to 8.44, p=0.02	1.69	0.58 to 4.92, p=0.34
	Other psychosis	139	66.9	1.20	0.82 to 1.76, p=0.34	0.99	0.65 to 1.52, p=0.96
Location	Inpatient	240	93.8	10.92	6.38 to 18.7, p<0.001	7.89	4.48 to 13.91, p<0.001
	Outpatient	947	57.9	Reference		Reference	

*Adjusted for age, gender, ethnicity, marital status, diagnosis, location and clinical service.

### Clinical outcomes

#### Number of inpatient days

The mean number of days which patients spent in hospital between 1 September 2014 and 28 February 2015 was 9.5 (SD 31.4). Those who had been approached for consent spent fewer days in hospital (mean 8.8, SD 29.5) than those who had not been approached (mean 9.6,SD 31.9). There was no significant difference between the two groups in univariate analysis (table 3, model 1), but multiple linear regression analysis (table 3, models 2–4) revealed a significant association between being approached for consent and spending fewer days in hospital, after adjusting for patient and mental health service-related factors. Within the group of patients who were approached, there was no significant association between whether consent was or was not given and the number of days spent in hospital (table 4).

**Table 3:** Association of being approached for consent with clinical outcome measures between 1 September 2014 and 28 February 2015, n=5787

	Number of inpatient days (n=5787)		Compulsory hospital admission (n=5787)		Change in total HoNOS score (n=3052)	
Model	β coefficient (days)	95% CI, p value	OR	95% CI, p value	β coefficient (per unit HoNOS)	95% CI, p value
1	−0.8	−2.8 to 1.2, p=0.41	0.82	0.65 to 1.04, p=0.10	−0.8	−1.4 to −0.3, p=0.003
2	−3.8	−5.8 to −1.8, p<0.001	0.65	0.51 to 0.83, p=0.001	−1.2	−1.7 to −0.6, p<0.001
3	−3.7	−5.6 to −1.7, p<0.001	0.64	0.50 to 0.82, p<0.001	−1.2	−1.8 to −0.7, p<0.001
4	−2.3	−4.4 to −0.3, p=0.03	0.65	0.50 to 0.84, p=0.001	−0.9	−1.5 to −0.4, p=0.001

Model 1: unadjusted.

Model 2: adjusted for number of hospital admissions between 1 September 2011 and 31 August 2014.

Model 3: adjusted for all factors in model 2 plus age, gender, ethnicity, marital status and diagnosis.

Model 4: adjusted for all factors in model 3 plus clinical service.

HoNOS, Health of the Nation Outcome Scale.

**Table 4:** Association of giving consent with clinical outcome measures between 1 September 2014 and 28 February 2015, n=1187

	Number of inpatient days (n=1187)		Compulsory hospital admission (n=1187)		Change in total HoNOS score (n=678)	
Model	β coefficient (days)	95% CI, p value	OR	95% CI, p value	β coefficient (per unit HoNOS)	95% CI, p value
1	4.3	0.8 to 7.9, p=0.02	1.38	0.87 to 2.20, p=0.17	−0.3	−1.3 to 0.7, p=0.58
2	2.4	−1.1 to 5.9, p=0.18	1.13	0.70 to 1.81, p=0.62	−0.7	−1.7 to 0.3, p=0.18
3	1.9	−1.6 to 5.4, p=0.30	1.10	0.67 to 1.79, p=0.72	−0.6	−1.7 to 0.4, p=0.23
4	1.6	−2.0 to 5.1, p=0.39	1.12	0.68 to 1.86, p=0.65	−0.6	−1.6 to 0.4, p=0.26

Model 1: unadjusted.

Model 2: adjusted for number of hospital admissions between 1 September 2011 and 31 August 2014.

Model 3: adjusted for all factors in model 2 plus age, gender, ethnicity, marital status and diagnosis.

Model 4: adjusted for all factors in model 3 plus clinical service.

HoNOS, Health of the Nation Outcome Scale.

#### Compulsory hospital admission under the UK Mental Health Act

The overall proportion of patients who were compulsorily admitted between 1 September 2014 and 28 February 2015 was 9.2%. Those who had been approached for consent were less likely to be compulsorily admitted (8.0%) than those who had not been approached (9.6%). There was no significant difference between the two groups in univariate analysis (table 3, model 1) but multiple linear regression analysis (table 3, models 2–4) revealed a significant association between being approached for consent and a reduced likelihood of compulsory admission, after adjusting for patient and mental health service-related factors. Among patients who had been approached, there was no significant association between whether consent had been given and the likelihood of compulsory admission (table 4).

#### Health of the Nation Outcome Scale

The overall mean total HoNOS score was 12.4 (SD 6.4). Those who had been approached for consent had a lower total HoNOS score (mean 11.7, SD 6.2) than those who had not been approached (mean 12.6, SD 6.5). Linear regression analysis (table 3) revealed a significant association between being approached and a lower total HoNOS score, both in univariate analysis (model 1) and in multiple linear regression analysis (models 2–4) adjusting for patient and mental health service-related factors. Among those approached, there was no significant association between whether consent was given and the mean total HoNOS score (table 4).

## Discussion

### Main findings

In this study, we investigated the proportion of patients with psychotic disorder who were willing to participate in research and assessed demographic and clinical factors that were associated with being approached for consent to be contacted to participate in research studies. We studied a large sample that is representative of people with psychotic disorders who receive care from inner city mental health services in the UK. We found that the majority of those approached were willing to be contacted in future about participation in research projects. Although univariate analyses suggested variation in the age, gender, ethnicity, marital status and diagnosis in relation to whether or not patients were approached for consent, multivariable analyses indicated that variation was largely related to mental health service-related factors than patient factors. An increase in number of previous hospital admissions was associated with an increased likelihood of being approached for consent. This might reflect the fact that patients who have multiple hospital admissions have greater opportunity to be approached for consent simply by virtue of more frequent contact with mental healthcare services with repeated opportunities to be approached for consent across multiple admissions. This is corroborated by the finding that patients were more likely to give consent if they were approached while in hospital (where there may have been multiple opportunities to discuss consent) rather than in the community.

We found that the patients who were approached for consent had fewer inpatients days, were less likely to be admitted to hospital compulsorily, and had lower HoNOS scores. It is possible that patients who appeared to be less severely unwell were more likely to be approached because the clinical staff perceived them as being more likely to tolerate participation in research. Furthermore, patients with severe illness may be perceived as lacking capacity to consent. Patients with a relatively good prognosis may have been more engaged with the clinical teams, and that this increased the likelihood of them being approached. We did not find any significant differences between patients who subsequently agreed to take part in research and those who did not. This may reflect the fact that clinician factors, such as how potential benefits and risks of being involved in clinical research are conveyed to patients or the enthusiasm of individual clinicians to encourage patients to be involved in research studies (which we were unable to measure in the present study), also have an important role in determining the likelihood of agreeing to participate in research. This is corroborated by the fact that adjusting for which clinical service a patient was receiving care from reduced or eliminated associations with individual patient factors seen in univariate analyses.

### Relationship to previous studies

We found a slightly lower level (65.1%) of consent than Callard *et al*^14^ who reported a 74.1% consent rate to participate in mental health research. However, they did not specifically study patients with psychotic disorders but all psychiatric disorders. Our results are in keeping with a recent study^11^ which found around 60% of those invited to participate in a research study agreed to do so. In a Canadian study of implementing a ‘permission to contact’ programme, Cheah *et al*^30^ reported overall consent rates ranging between 80% (congenital heart disease) and 90% (cancer). This may echo the challenges of recruiting patients with psychotic disorders into clinical studies. A possible explanation for reduced rates of consent in people with psychotic disorders is degree of insight into the illness and perceived need for treatment. Some patients with a psychotic disorder do not believe they are unwell or require treatment and may therefore not believe in the need for research into the disorder.^31^ Other factors associated with research participation such as timing of the approach and communication skills may also explain this variation.^31^

We found that individuals of black ethnic group were less likely to give consent, in keeping with previous studies.^3^ ^12^ ^32^ Conversely, individuals of ‘other’ ethnic group were also less likely to be approached independent of potential confounders, a finding also reported in other studies.^13^ ^33^ Furthermore, our results showed that younger patients were more likely to be approached for consent, consistent with a previous study.^2^ One explanation for this finding might be that mental healthcare professionals believe that younger patients are more likely to be interested in participating in mental health research than older patients. However, our results also indicated that older individuals were less likely to give consent independent of potential confounders, as previously reported findings both in mental and physical healthcare research.^14^ ^33^

In addition, our results showed that only 20% of the sample was approached for (C4C) and that patients in hospital were more likely to be approached than patients in the community. This may be explained by resource issues as evidence suggests that clinicians struggle with resources and support to devote time to research.^34^ ^35^ Another possible contributory factor is the challenges of recruiting patients with diagnosis of psychotic disorder into clinical research.^2^ ^17^

### Strengths

The extent to which patients are willing to participate in clinical research has already been established in other areas of medicine such as oncology and cardiology.^30^ ^36^ To the best of our knowledge, the present study is the first to examine this issue in patients with psychotic disorders, and did this in a large sample that is representative of the population of patients that are seen by inner city mental health services.

### Limitations

The limitations of this study include its cross-sectional observational design. Therefore, causality cannot be inferred from the association of being approached for consent (or giving consent when approached) with clinical outcomes. As discussed previously, there are a number of possible explanations for the associations of consent with clinical outcomes and it is likely that these are related to a complex set of patient and healthcare service-related factors. There is a complex and multifactorial relationship between the factors analysed in this study and the likelihood of being approached for consent and giving consent. While we adjusted for individual characteristics and service-related factors, our study could still be confounded by unmeasured characteristics of the services that were more likely to approach patients for consent. It could be that these services have enthusiasm and sustained research culture embedded in their practice thus making them more likely to discuss consent with their patients. Although we have assumed that all persons receiving care within SLaM would be asked about consent, our study may still suffer selection bias as clinicians may have approached more settled and higher functioning patients who may be more likely to say yes to being approached for research. Patients may also be more likely to be approached in clinical areas where C4C implementation team is engaged. In addition, only around 50% of patients had complete HoNOS data available and so it is possible that the associations of C4C with HoNOS may not have accurately represented the whole population. Furthermore, in this cross-sectional study it was not possible to examine change in HoNOS score which may have occurred during the course of receiving mental healthcare.

### Future research

The results of the current study highlight some important areas for further investigation. For instance, to determine why there were significant differences in the likelihood of being approached depending on patient and mental health service-related factors. The decision to participate in research may also partly depend on how a potential participant is approached to gain consent by individual clinicians. Future studies investigating patient and clinician factors may help to determine how patients with psychotic disorders may better engage with clinical research opportunities.

## Conclusions

Most patients with psychosis were willing to be approached about participation in research but only a small proportion of patients were approached for consent. Those who were approached tended to be patients who were less severely unwell and had relatively good clinical outcomes: this may reflect a bias of staff towards considering less unwell patients for research. Clinician factors may also be important in determining likelihood of giving consent to participate in research and further work is needed to increase research participation among individuals with psychotic disorders.
